# In praise of Prais‐Winsten: An evaluation of methods used to account for autocorrelation in interrupted time series

**DOI:** 10.1002/sim.9669

**Published:** 2023-02-01

**Authors:** C Bottomley, M Ooko, A Gasparrini, RH Keogh

**Affiliations:** ^1^ London School of Tropical Medicine & Hygiene MRC International Statistics and Epidemiology Group London UK; ^2^ Department of Infectious Disease Epidemiology London School of Hygiene & Tropical Medicine London UK; ^3^ Department of Epidemiology and Demography Kemri‐Wellcome Trust Research Programme Kilifi Kenya; ^4^ Department of Public Health, Environments and Society London School of Hygiene and Tropical Medicine London UK; ^5^ Centre for Statistical Methodology London School of Hygiene and Tropical Medicine London UK; ^6^ Department of Medical Statistics London School of Hygiene and Tropical Medicine London UK

**Keywords:** autocorrelation, interrupted time series, intervention analysis

## Abstract

Interrupted time series are increasingly being used to assess the population impact of public health interventions. These data are usually correlated over time (auto correlated) and this must be accounted for in the analysis. Typically, this is done using either the Prais‐Winsten method, the Newey‐West method, or autoregressive‐moving‐average (ARMA) modeling. In this paper, we illustrate these methods via a study of pneumococcal vaccine introduction and explore their performance under 20 simulated autocorrelation scenarios with sample sizes ranging between 20 and 300. We show that in terms of mean square error, the Prais‐Winsten and ARMA methods perform best, while in terms of coverage the Prais‐Winsten method generally performs better than other methods. All three methods are unbiased. As well as having good statistical properties, the Prais‐Winsten method is attractive because it is decision‐free and produces a single measure of autocorrelation that can be compared between studies and used to guide sample size calculations. We would therefore encourage analysts to consider using this simple method to analyze interrupted time series.

## 1 INTRODUCTION

An interrupted time series (ITS) consists of observations made before and after an event of interest that are used to assess its impact. The event may be planned, such as the introduction of a vaccination program, or unplanned such as the 2008 global financial crisis or recent COVID‐19 pandemic.^1^ In either case, a defining feature of this study design is that the observations are made at the population level. ITS analyses therefore assess the population‐level impact of an event. For example, we may be interested in estimating the difference in the mean disease incidence following the introduction of a vaccine vs what it would have been had the vaccine not been introduced.

The impact of the event can be estimated by fitting a regression model to compare the pre‐ and post‐event periods, adjusting for confounding due to time trend and seasonality where necessary. A problem with this approach is that the usual independence assumption is difficult to justify when the residuals are correlated in time, as is often the case in ITS analyses. Typically, residuals that are close in time are more similar than those that are further apart. This so‐called autocorrelation must be accounted for otherwise the analysis will produce incorrect—usually anticonservative—*p*‐values and confidence intervals.^2^

Three approaches are commonly used to account for autocorrelation in ITS. The first is to assume the residuals follow a first‐order autoregressive process and fit the regression model using the Prais‐Winsten procedure.^3^ As a form of generalized least squares (GLS), the Prais‐Winsten method works by applying a linear transformation to the outcome and explanatory variables in order to decorrelate the error term. Because a first‐order autoregressive error model is assumed, the appropriate linear transformation is determined by a single parameter representing the correlation between residuals at consecutive time points. The second approach is to fit several autoregressive‐moving‐average (ARMA) models by maximum likelihood, and then use either the autocorrelation function or a statistical criterion, such as the AIC, to choose the best‐fitting model.^4^, ^5^ Finally, the third approach is to ignore autocorrelation in the estimation of the regression parameters and adjust the standard errors using the Newey‐West method.^6^ This approach is essentially an extension of the robust standard errors methodology that is commonly used to adjust for clustering and heteroskedasticity.

Here we conduct a simulation study to evaluate these different methods under a range of autocorrelation scenarios. In this evaluation, we assume the ARMA error model is unknown and consider the selection of an appropriate model as part of the estimation procedure. The study builds on previous simulation studies where an order‐1 autoregressive model has been assumed.^7^, ^8^, ^9^, ^10^ Our main finding is that the Prais‐Winsten method generally has coverage closer to the nominal value than other methods.

The paper is structured as follows. We begin by describing a regression model that is widely used to analyze ITS and three methods commonly used to account for autocorrelation (Sections 2 and 3). The methods are illustrated using data from a pneumococcal vaccine impact study (Section 7). We then present a simulation study to evaluate the methods in terms of bias, mean square error, and confidence interval coverage (Section 5). Results from the simulation study show marked differences in coverage, with the Prais‐Winsten method generally having better coverage than other methods. In response to this finding, we explore reasons for the observed variation and approaches that can be used to bring coverage closer to the nominal level (Section 6). We also briefly explore the issue of statistical power. Finally, we conclude with a discussion of our findings (Section 7).

## 2 MODELLING ITS

An ITS consists of a number of measurements, such as the number of cases of disease, made on a population before and after an event of interest. We let $y_{t}$ (t=0,…,n−1) denote observations of the outcome at n equally spaced times, and τ denote the time of the event, which for concreteness we assume is an intervention rather than an unplanned event. A simple model for the ITS $y_{t}$ (t=0,…,n−1) is:

(1) $$y_{t}=\beta_{0}+\beta_{1}x_{t}+\varepsilon_{t},$$

where $x_{t}$ represents an indicator for the intervention ($x_{t}=0$ for t<τ and $x_{t}=1$ for t≥τ), and $\varepsilon_{t}$ is a mean zero error that represents other determinants of the outcome. Assuming $x_{t}$ is independent of $\varepsilon_{t}$, that is assuming no confounding, an unbiased estimate of the intervention effect, $\beta_{1}$, can be obtained by regressing $y_{t}$ on $x_{t}$.

In practice, it is often necessary to adapt this basic model. In particular, the model must be modified when the “other determinants” include confounding factors that are correlated with $x_{t}$. If such factors are ignored in the regression, then the resulting estimate of $\beta_{1}$ is no longer an unbiased estimate of the intervention effect. Graphically, confounding manifests itself as a trend in $y_{t}$ (unless the confounding factors are perfectly correlated with $x_{t}$). Thus, approaches for dealing with confounding often involve modeling trend rather than modeling confounder effects directly.^11^ The trend can be modeled as linear, non‐linear, or stochastic.^12^

The simplest approach, which is frequently used in the medical literature and is often reasonable for modeling short time series, is to assume that the confounding can be controlled via a linear trend term. This is the so‐called segmented regression model:

(2) $$y_{t}=\beta_{0}+\beta_{1}x_{t}+\beta_{2}t+\varepsilon_{t}.$$

The model sometimes also includes an interaction between $x_{t}$ and t. Usually the interaction term is interpreted as a changing intervention effect but it could equally represent a non‐linear trend.

The model in Equation (2) can be estimated using ordinary least squares regression (OLS). However, a problem with using OLS is that the resulting *p*‐values and confidence intervals are only valid if the $\varepsilon_{t}$ are mutually independent. This assumption usually does not hold for time series data since the residuals tend to be positively correlated. In this situation, OLS produces a standard error SE that is downward‐biased^2^ and, as a result, the confidence interval and *p*‐value for the intervention effect are anti‐conservative. In the next section, we describe three methods commonly used to account for autocorrelation in ITS analyses.

## 3 METHODS USED TO ACCOUNT FOR AUTOCORRELATION

### 3.1 Prais‐Winsten

The Prais‐Winsten method involves estimating the correlation between the error at t and t−1, $\text{corr}\left(\epsilon_{t},\epsilon_{t-1}\right)$, and then using this estimate to transform the outcome and predictor variables in such a way that the correlation is removed from the error when a linear regression model is fitted to the transformed data. The key assumption behind the method is that the error follows a first‐order autoregressive process; autocorrelation is therefore only fully removed if the error follows this model. The method is an example of feasible generalized least squares and, as such, produces estimates with the same asymptotic distribution as the maximum likelihood estimator—see, for example, chap. 8 in Hamilton's textbook.^13^ The following outline is based on the description presented by Woodridge.^2^

We assume the no‐trend model (Equation 1) in which the error follows a first‐order autoregressive process. Specifically, we assume that

(3) $$\epsilon_{t}=\phi_{1}\epsilon_{t-1}+\eta_{t},$$

where $|\phi_{1}|<1$ and $\eta_{t}$ are independent disturbances with zero mean and variance $\sigma^{2}$. The constraint on the autoregressive (AR) parameter $\phi_{1}$ ensures the process is stationary, that is, $\mathrm{cov}(\epsilon_{t}$, $\epsilon_{t+h})$ is independent of t, and therefore that the variance remains constant over time.

If we also assume that $\phi_{1}$ is known, then we can remove the correlation in the errors by applying the transformation ${\tilde{y}}_{t}=y_{t}-\phi_{1}y_{t-1}$ and ${\tilde{x}}_{t}=x_{t}-\phi_{1}x_{t-1}$ for t=1,…,n−1. In terms of the transformed data, Equation (1) becomes:

(4) $${\tilde{y}}_{t}=\beta_{0}\left(1-\phi_{1}\right)+\beta_{1}{\tilde{x}}_{t}+\eta_{t},$$

where the errors, $\eta_{t},$ are now mutually independent. Hence the intervention effect, $\beta_{1}$, can be estimated by defining a constant predictor $z_{t}=\left(1-\phi_{1}\right)$ and regressing ${\tilde{y}}_{t}$ on ${\tilde{x}}_{t}$ and $z_{t}$ in a model without an intercept term.

Because $\;\phi_{1}$ is usually unknown, $y_{t}$ and $x_{t}$ must be transformed using an estimate of this parameter, that is, ${\tilde{y}}_{t}=y_{t}-{\hat{\phi }}_{1}y_{t-1}$ and ${\tilde{x}}_{t}=x_{t}-{\hat{\phi }}_{1}x_{t-1}$. Typically, ${\hat{\phi }}_{1}$ is the estimated slope parameter from the regression of ${\hat{\epsilon }}_{t}$ on ${\hat{\epsilon }}_{t-1}$ where these residuals are obtained from the regression of $y_{t}$ on $x_{t}$ (Equation 1). The regression of ${\hat{\epsilon }}_{t}$ on ${\hat{\epsilon }}_{t-1}$ can be conducted with or without an intercept; either way, the regression coefficient for ${\hat{\epsilon }}_{t-1}$ provides a consistent estimate of $\phi_{1}$.

The above method is referred to as the Cochrane‐Orcutt method.^14^ The Prais‐Winsten method^3^ is an extension of this method that includes $y_{0}$ and $x_{0}$ in the analysis and thereby increases the precision of the parameter estimates. To ensure that the error variance is independent of time, $y_{0}$ and $x_{0}$ are scaled by the factor $\sqrt{\left(1-{\hat{\phi }}_{1}^{2}\right)}$, that is ${\tilde{y}}_{0}=y_{0}\sqrt{\left(1-{\hat{\phi }}_{1}^{2}\right)}$ and $\;{\tilde{x}}_{0}=x_{0}\sqrt{\left(1-{\hat{\phi }}_{1}^{2}\right)}$. Then, as in the Cochrane‐Orcutt method, ${\tilde{y}}_{t}$ is regressed on ${\tilde{x}}_{t}$ and $z_{t}$, where $z_{t}=\sqrt{\left(1-{\hat{\phi }}_{1}^{2}\right)}$ for t=0 and $z_{t}=\left(1-\phi_{1}\right)$ for t>0. As in the Cochrane‐Orcutt method, the regression is fitted without an intercept term.

Additional covariates can be handled similarly. For example, to fit the model with trend (Equation 2)—and assuming first‐order autoregressive error—we would need to transform t in addition to $x_{t}$ and $\;y_{t}$.

### 3.2 Auto‐regressive‐moving‐average

The first‐order autoregressive error model described above (Equation 3) is a special case of an auto‐regressive‐moving‐average (ARMA) model. In this more general model, the error at time t depends on the errors at the p most recent time points (AR part of the model) and q disturbance terms (MA part of the model), that is:

(5) $$\epsilon_{t}=\phi_{1}\epsilon_{t-1}+\cdots +\phi_{p}\epsilon_{t-p}+\theta_{1}\eta_{t-1}+\cdots +\theta_{q}\eta_{t-q}+\eta_{t},$$

where the disturbances $\eta_{t}$ (also called innovations) are assumed to be uncorrelated and normally distributed with zero mean and constant variance $\sigma^{2}$.

Assuming an ARMA error, the likelihood of the data can be written as $\prod_{t=0}^{n}\;f\left(y_{t}|y_{t-1},\ldots ,y_{0};\zeta \right)$ and model parameters ζ estimated by maximising this likelihood. Note that ζ includes both the parameters from the ARMA error model and from the model of the mean. The two approaches most commonly used to implement maximum likelihood estimation are: (1) to use the Kalman filter to maximize the full likelihood and (2) to maximise a conditional likelihood obtained by fixing $\eta_{p-1},\ldots ,\eta_{p-q}$ at zero and $\epsilon_{0},\ldots .,\epsilon_{p-1}$ at their observed values (ie, the residuals at these time points). The two approaches are described in Hamilton's textbook.^13^ As an illustration of the conditional likelihood approach, consider the no trend model (Equation 1) with MA(1) error, that is, $\zeta =\left(\beta_{0},\beta_{1},\theta_{1},\sigma^{2}\right)$. If we set $\eta_{-1}=0$ then $\epsilon_{0}=\eta_{0}$, $\epsilon_{1}=\theta_{1}\epsilon_{0}+\eta_{1}$ and $\epsilon_{2}=\theta_{1}\left(\epsilon_{1}-\theta_{1}\epsilon_{0}\right)+\eta_{2}$. Because $\epsilon_{t}=y_{t}-\beta_{0}-\beta_{1}x_{t}$, the first three terms of the likelihood are $y_{0}∼N\left(\beta_{0}+\beta_{1}x_{0},\;\sigma^{2}\right)$, $y_{1}|\;y_{0}∼N\left(\beta_{0}+\beta_{1}x_{1}+\theta_{1}\epsilon_{0},\;\sigma^{2}\right)$ and $\;y_{2}|y_{1},y_{0}∼N\left(\beta_{0}+\beta_{1}x_{2}+\theta_{1}\left(\epsilon_{1}-\theta_{1}\epsilon_{0}\right),\;\sigma^{2}\right)$, and the subsequent terms can be derived by further iterating the error equation.

To choose the form of the ARMA error model—that is, the values of p and q —some authors recommend inspecting the autocorrelation function and partial autocorrelation functions of the residuals.^5^ For example, zero autocorrelation beyond lag 1 implies an MA(1) model (ie, an ARMA model with p=0 and q=1). Others recommend fitting a number of different ARMA models and using a statistical criterion like the AIC, AICc or BIC to select the best fitting model.^4^ This approach is appealing because it reduces subjectivity. Indeed, in an early paper on the AIC, Akaike argued for using the criteria to “relieve the time series analyst of much of the burden of making subjective judgements”.^15^ A drawback is that it is often unclear how many models should be used in the comparison. One strategy for dealing with this problem is to use a forward selection algorithm in which p and q are increased incrementally until there is no further improvement in model fit as measured by AIC, for example.^16^

### 3.3 Newey‐West

The OLS estimate of the intervention effect is unbiased provided the model for the mean is correctly specified, even in the presence of autocorrelation. Thus, another way to deal with autocorrelation is to use the OLS estimate of the intervention effect and adjust the SE. The Newey‐West method does exactly this.^6^ It uses the observed correlation between residuals to produce a so‐called robust SE. The method is closely related to the methods proposed by White and Liang and Zeger to account for heteroskedasticity and clustering.^17^, ^18^

The key assumption of the Newey‐West method is that the error correlation is zero beyond a certain lag m. It is therefore tempting to use a large value of m to minimize the impact of this assumption. Unfortunately, however, the variance estimate is only consistent if m is small relative to the number of observations (n).^6^ So how should m be chosen? One option is to use the integer part of $n^{1/4}$. This rule is motivated by the fact that in the original paper by Newey and West one of the conditions used to prove consistency was that the rate of increase in m should be slower than $n^{1/4}.$ ^2^ Alternatively, several data‐dependent strategies have also been proposed.^19^, ^20^ A simplification of one of these, assuming an AR(1) autocorrelation model with correlation parameter 0.25, leads to the rule $m=0.75\;n^{1/3}.$ ^20^ More recently it has been shown that size distortion in hypothesis testing may be further reduced using the rule $m=1.3\;n^{1/2}$ in conjunction with fixed‐b critical values.^21^

## 4 EXAMPLE: INTRODUCTION OF A PNEUMOCOCCAL VACCINE IN KENYA

**FIGURE 1:**
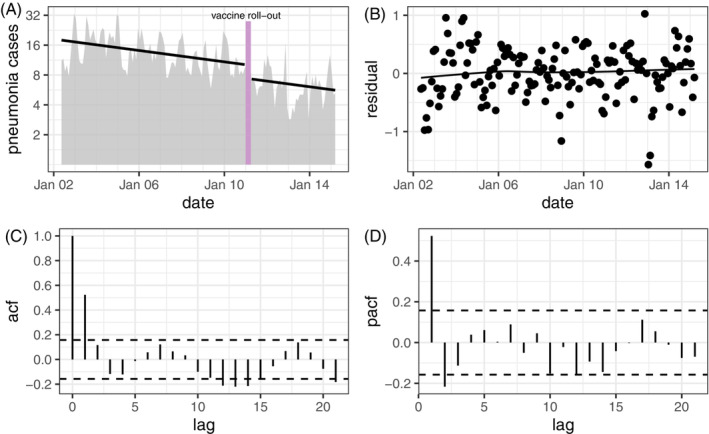
(A) Monthly incidence (per 10 000) of severe and very severe clinical pneumonia in children <5 years before and after the introduction of pneumococcal vaccination in Kilifi, Kenya. The solid black line represents the trend and vaccine impact estimated by fitting a linear regression model to the data (Prais‐Winsten and ARMA model produced similar estimates). The vertical bar represents the period of vaccine roll‐out (Jan ‐ Mar 2011). (B) Residuals with loess trend line. (C) Autocorrelation function for the residuals. (D) Partial autocorrelation function for the residuals

To illustrate the three different methods outlined above, we use data from an ITS study of the impact of the introduction of 10‐valent pneumococcal vaccine (PCV10) on severe and very severe clinical pneumonia in Kenya.^22^ The data consist of monthly hospital admissions for severe or very severe pneumonia in children <5 years collected over a period of 155 months (104 months pre‐vaccine introduction and 51 months post introduction) between May 2002 and March 2015 (Figure 1A).

In the original analysis, an AR(2) model was selected based on a comparison of AIC among all ARMA models with p≤3 and q≤3. Here we present a reanalysis of these data using the three methods described in Section 3. In each case, we fitted a segmented regression model (Equation 2) to the log_2_‐transformed incidence rates. A plot of the residuals vs study month suggests that the linear trend assumption is reasonable for these data (Figure 1B). In addition to terms for trend and post‐vaccine period (Jan 2011 ‐ Mar 2015), the model included: (i) a binary indicator for health worker strikes (ii) a categorical variable for calendar month and (iii) an indicator for the vaccine roll out period (Jan ‐ Mar 2011). Calendar month was included to account for seasonality in pneumonia incidence and the vaccine rollout period was included to exclude this period from intervention effect estimate.

All analyses were done using R version 3.6.1.^23^ The ARMA model fitting and selection was done using the *auto.arima* function (package = *forecast*).^16^ Specifically, we used *auto.arima* to implement stepwise selection based on a bias corrected version of the AIC, as recommended by Hyndman and Athanasopoulos,^4^ with the constraint that *p* ≤ 5 and *q* ≤ 5. The Newey‐West method was implemented using the *NeweyWest* function (package = *sandwich*)^24^ with m chosen according to the method described by Newey and West.^19^ Finally, the Prais‐Winsten method was implemented using the *prais.winsten* function (package = *prais*).^25^ Table 1 shows the intervention effect estimate and confidence interval generated by each method together with the OLS estimate and confidence interval (unadjusted for autocorrelation). The data and code for these analyses are available at https://github.com/christian‐bottomley/ITS_Autocorrelation.

**TABLE 1:** Estimates of the trend in pneumonia incidence and vaccine impact

Method	Trend (% reduction per month)	95% CI	Vaccine impact (% reduction)	95% CI
OLS	0.55	0.35, 0.74	27.0	11.5, 39.8
Prais‐Winsten	0.56	0.22, 0.89	26.4	−2.2, 47.0
ARMA^a^	0.54	0.26, 0.82	27.9	5.1, 45.2
Newey‐West^b^	0.55	0.25, 0.84	27.0	6.4, 43.1

^a^ ARMA model with *p* = 0 and *q* = 2.

^b^ Accounting for autocorrelation up to lag 9.

The point estimates are similar across all the methods suggesting that the vaccine reduces the incidence of severe and very severe pneumonia by about 27%. The OLS 95% confidence interval is narrower than the other confidence intervals which is unsurprising since there is strong evidence of autocorrelation (OLS: 11.5, 39.8, Prais‐Winsten: −2.2, 47.0; ARMA: 5.1, 45.2; Newey‐West: 6.4, 43.1). Figure 1C shows that the autocorrelation is strongest at lag 1 and Figure 1D, which shows the partial autocorrelation function, suggests an AR(2) model might be appropriate. Among the methods that account for autocorrelation, the Newey‐West and ARMA confidence intervals are similar but the Prais‐Winsten confidence interval is significantly wider. However, it is not obvious which is most appropriate—all of them account for significant lag‐1 autocorrelation, which is the main feature of these data. This ITS analysis is not unusual in being sensitive to the choice of method. In an empirical evaluation of different methods for analyzing ITS, including Prais‐Winsten and Newey‐West, Turner et al found that statistical significance (*p* < 0.05) differed in 4 to 25% of the pair‐wise comparisons.^26^

## 5 SIMULATION

We assessed the performance of the different methods by simulating data from the segmented regression model (Equation 2) under 20 different autocorrelation scenarios and 4 different scenarios for ITS length (*n* = 20, 50, 100 and 300). To simulate from the model, we used a 1:1 ratio for the numbers of observations before and after the intervention and fixed $\beta_{0}=4,\;\beta_{1}=-1$ and $\beta_{2}=-1/n$ based on the relative reductions associated with intervention (25%) and trend (25%) in the pneumonia example. We note, however, that inference should be unaffected by the choice of parameter values because the SE is independent of the parameter values of the regression.^27^ Our own experience of using different values and sensitivity analyses conducted in previous simulation studies also suggest that our findings are independent of the chosen regression parameter values.^7^

We assumed an MA(3) model for the error, that is we assumed $\epsilon_{t}=\theta_{1}\eta_{t-1}+\theta_{2}\eta_{t-2}+\theta_{3}\eta_{t-3}+\eta_{t}$ with Var $(\eta_{t}$) = 1. This model allows for an arbitrary correlation structure up to lag 3 but assumes zero correlation beyond this point. The 20 autocorrelation scenarios were chosen by randomly sampling $\theta_{1}$ from unif(0, 1), $\theta_{2}$ from unif(0, $\theta_{1})$ and $\theta_{3}$ from unif(0, $\theta_{2})$. By selecting the parameters in this way, the autocorrelation was constrained to be positive and decreasing over time.

For each scenario, 2000 datasets were generated. Intervention effect estimates (ie, estimates of $\beta_{1})$ and 95% confidence intervals were obtained by implementing the methods as in the pneumococcal vaccine example, and their performance was evaluated in terms of bias, mean square error and coverage of 95% confidence intervals using the rsimsum package.^28^ We also evaluated estimates obtained by fitting the true MA(3) model via maximum likelihood. The code for the simulation study and a complete table of results, including Monte Carlo error estimates, is available at https://github.com/christian‐bottomley/ITS_Autocorrelation.

### 5.1 Simulation results

**FIGURE 2:**
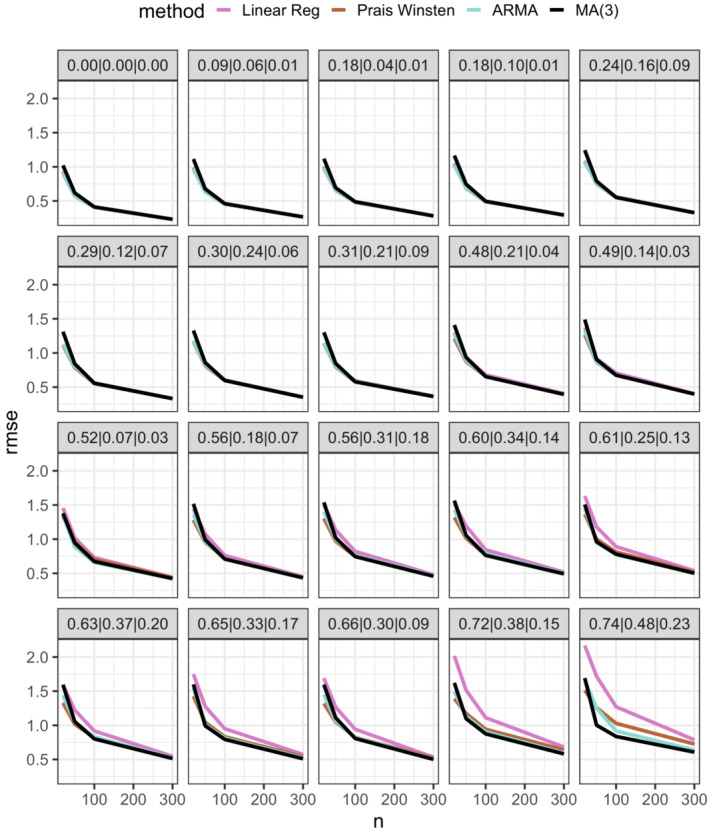
Root mean square error (RMSE) as a function of ITS length (*n*) in 20 autocorrelation scenarios. The scenarios range from lowest correlation in the top left (lag‐1, lag‐2 and lag‐3 correlations of 0.06, 0.02 and 0.01 respectively) to highest correlation in bottom right (lag‐1, lag‐2 and lag‐3 correlations of 0.74, 0.48 and 0.23 respectively)

**FIGURE 3:**
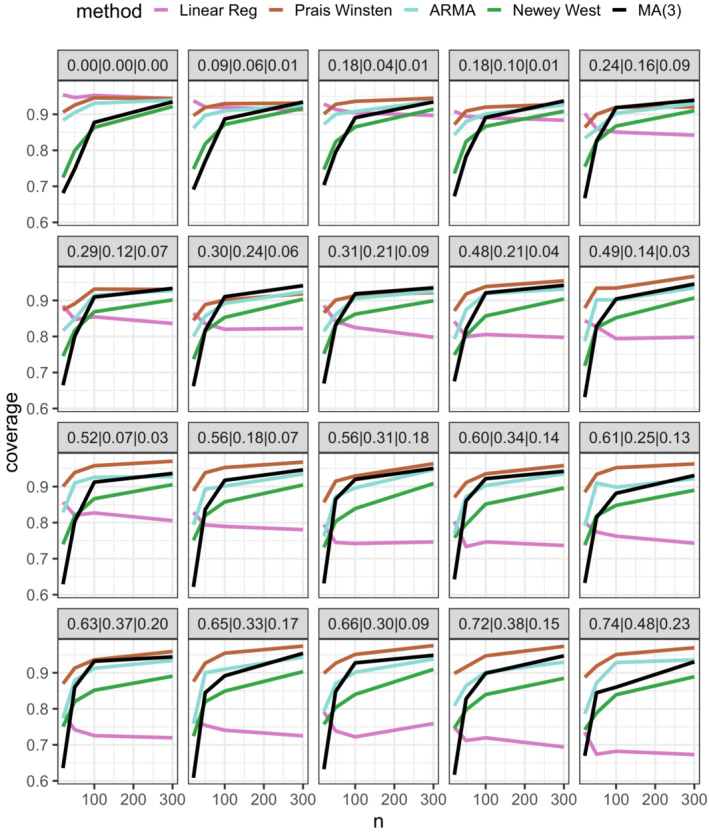
Confidence interval coverage (nominal value = 0.95) as a function of ITS length in 20 autocorrelation scenarios. The scenarios range from lowest correlation in the top left (lag‐1, lag‐2 and lag‐3 correlations of 0.06, 0.02 and 0.01 respectively) to highest correlation in bottom right (lag‐1, lag‐2 and lag‐3 correlations of 0.74, 0.48 and 0.23 respectively)

The results from the simulation study are summarized in Supplementary Figure 1 (bias), Figure 2 (root mean square error) and Figure 3 (coverage) and in the text below.

*Bias*: It is well known that OLS estimates are unbiased even when errors are correlated.^13^ Moreover, Prais‐Winsten and ARMA estimates are also unbiased under very general conditions, including correlated errors, because they can be viewed as feasible generalized least squares estimates.^29^ Our simulations results are consistent with these theoretical results. Across all sample size and autocorrelation scenarios, the mean bias was close to zero (OLS & Newey‐West = −0.0039, Prais‐Winsten = −0.0031, ARMA = −0.0033, MA(3) = −0.0024). Furthermore, in 76 out of the 80 scenarios, the 95% Monte Carlo confidence interval for the bias estimate included zero, irrespective of the method.

*Mean square error*: In scenarios where the degree of autocorrelation was low to moderate (lag‐1 correlation <0.6) the different methods performed similarly in terms of MSE. The mean root MSE (across all sample size scenarios) was: OLS & Newey‐West = 0.75, Prais‐Winsten = 0.72, ARMA = 0.73, MA(3) = 0.77. In the high autocorrelation scenarios (lag‐1 correlation ≥0.6) the Prais‐Winsten and ARMA methods performed significantly better than OLS (mean root MSE: OLS & Newey‐West = 1.17, Prais‐Winsten = 0.97, ARMA = 0.99, MA(3) = 0.99).

*Coverage*: In general, coverage was below the nominal 95% level; however, there was significant variation between the methods. Across all scenarios, the mean coverage was: OLS = 81.3%, Prais‐Winsten = 92.2%, ARMA = 88.3%, Newey‐West = 82.9% and MA(3) = 82.9%. The variation in performance was particularly apparent in scenarios with n≤50 (mean coverage: OLS = 84.3%, Prais‐Winsten = 88.0%, ARMA = 80.8%, Newey‐West = 74.3%, MA(3) = 65.2%). In these scenarios, coverage was generally worst when an MA(3) model (the true model) was fitted and best when the Prais‐Winsten method was used, suggesting an inverse relationship between coverage and number of parameters included in the error model. In a supplementary analysis exploring the coverage of different MA models, we also observed an inverse relationship between coverage and number of parameters (Supplementary Figure 2). In scenarios with n≤50 and low levels of autocorrelation (lag‐1 correlation <0.3), OLS produced coverage that was slightly closer to the nominal level than the Prais‐Winsten method (mean coverage: OLS = 90.8%, Prais‐Winsten = 89.9%).

## 6 COVERAGE AND POWER

In our simulation study, we found that under coverage was pervasive, particularly when error models with multiple autocorrelation parameters were fitted. Here we briefly discuss the issue and possible solutions. We also show that complex error models are associated with reduced statistical power.

### 6.1 Coverage

Confidence intervals for the intervention effect estimate, ${\hat{\beta }}_{1},$ are based on the distribution of the test statistic

$$T=\frac{{\hat{\beta }}_{1}-\beta_{1}}{\hat{\mathrm{se}}\left({\hat{\beta }}_{1}\right)\;}$$

where $\hat{\mathrm{se}}\left({\hat{\beta }}_{1}\right)$ is an estimate of the SE of ${\hat{\beta }}_{1}$. Typically, it is assumed that the distribution of T can be approximated by a standard normal distribution and 95% confidence intervals take the form ${\hat{\beta }}_{1}\pm 1.96\;\hat{\mathrm{se}}\left({\hat{\beta }}_{1}\right)$.

In the hypothetical scenario where the error follows an ARMA model with known parameters, the estimated SE can be replaced by the *known* SE and T follows a standard normal distribution exactly. In this situation, confidence intervals based on this distribution are guaranteed to have correct coverage.

**FIGURE 4:**
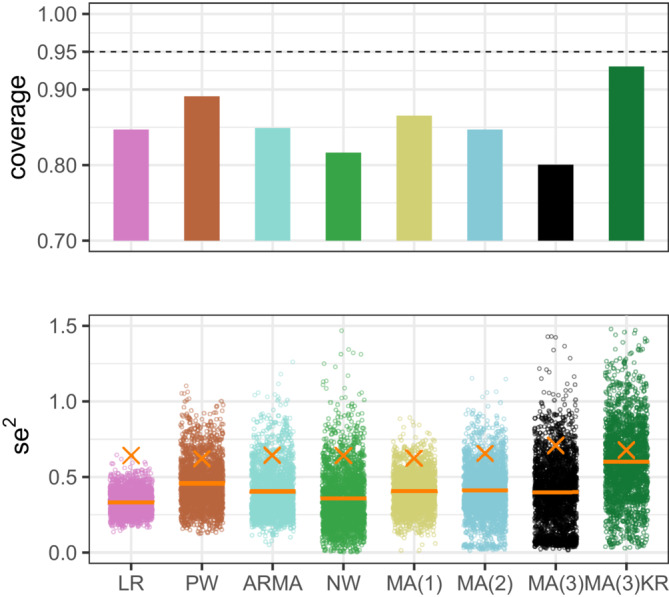
Distribution of estimates of $\mathrm{se}{\left({\hat{\beta }}_{1}\right)}^{2}$ and coverage under moderate autocorrelation (lag 1 = 0.29, lag 2 = 0.12, lag 3 = 0.07) and *n* = 50. The true value is denoted by a cross and horizontal bars represent the median of the distribution. Downward bias and variability in these estimates reduce the coverage of confidence intervals based on a standard normal distribution. The amount of bias differs between methods, as does the amount of variability. In particular the variability increases as the number of parameters included in the MA error model increases from 1 to 3. The coverage can be brought close to the nominal value by using the Kenward Roger (KR) method, which reduces bias and accounts for variability by basing confidence intervals on a t‐distribution rather than the standard normal

In practice, however, the parameters of the error model are unknown and an estimate of the SE must be used. This leads to under coverage for two reasons: (1) the SE is underestimated because of bias in the error model parameter estimates due to overfitting (2) variability in the SE estimate is unaccounted for. Variation and bias in the SE estimates are illustrated in Figure 4. In this figure, we see that differences between methods in these characteristics translate into differences in coverage. In particular, the Prais‐Winsten method achieves coverage closest to the nominal value (among the standard methods) because both bias and variation are kept low. We use the MA models in the figure to illustrate the effect of increasing the number of model parameters. Consistent with our earlier observation that coverage decreases with increasing model complexity, here we see that both bias and variability increase with increasing model complexity.

Bias in the SE can be reduced by using restricted maximum likelihood estimation (REML) instead of maximum likelihood estimation, an approach commonly used in mixed effects modeling.^30^, ^31^ The idea behind REML is to linearly transform the outcome vector so that the likelihood is a function of the error model parameters only. Once the error model parameters have been estimated by maximizing this restricted likelihood, the regression parameters can be estimated by maximizing the full likelihood with the error model parameters fixed at their estimated values. Computationally the estimation of the regression parameters is equivalent to generalized least squares. By making the error model estimation independent of the parameters of the regression model, REML reduces bias in the estimation of the error model, which, in turn, reduces bias in the SE estimates (Supplementary Figure 3). At present, REML is rarely used to analyze time series, though several authors have argued that it is useful in this setting.^10^, ^26^, ^27^, ^32^

To further improve coverage, several authors^33^, ^34^, ^35^ have proposed assuming $\hat{\mathrm{var}}\left({\hat{\beta }}_{1}\right)$ follows a scaled chi‐square distribution and using Satterthwaite's approximation^36^ to compute the appropriate degrees of freedom. By making this assumption, T is approximated by a t‐distribution rather than the standard normal. Specifically, it is assumed $\left(\frac{d}{\phi }\right)\;\hat{\phi }∼\chi_{d}^{2}$, where $\phi =\mathrm{var}\left({\hat{\beta }}_{1}\right)$ and $\hat{\phi }$ is the estimate of ϕ. The degrees of freedom are estimated from the expression $d=\frac{2\;\phi^{2}}{\mathrm{var}\left(\hat{\phi }\right)}$, which is derived by matching variances, and plugging in appropriate estimates of ϕ and $\mathrm{var}\left(\hat{\phi }\right)$ ($\hat{\phi }$ is used to estimate ϕ and an estimate for $\mathrm{var}\left(\hat{\phi }\right)$ can be obtained via a Taylor series approximation). The Satterthwaite approximation can be used with either maximum likelihood or REML but since the aim is to improve coverage it is generally used with REML. Kenward and Roger suggest making a further correction to the SE, which slightly improves coverage.^31^, ^35^

In Supplementary Figure 2, the coverage of MA(3) fitted via maximum likelihood is compared with coverage of MA(3) fitted via REML with the Kenward‐Roger adjustments. It can be seen that the Kenward‐Roger method largely solves the problem of low coverage.

### 6.2 Power

To achieve correct coverage, the degrees of freedom must be significantly reduced when an MA(3) model is fitted even when there is no autocorrelation. This suggests that fitting an over‐parameterized error model comes at a cost in terms of statistical power. For example, in the zero‐autocorrelation scenario with *n* = 50, on average 9.4 degrees of freedom are used in the Kenward‐Roger adjustment compared with 47 in OLS, which translates into a difference in power of 42% vs 33%. Unfortunately, it is difficult to compare power more widely because of differences in coverage. In Supplementary Figure 4, we limit the influence of coverage by restricting the comparison to methods with coverage >85%. In this analysis, MA(3) with Kenward‐Roger adjustment has lower power than other methods, though at high levels of autocorrelation the comparison is only between Prais‐Winsten and MA(3) with Kenward‐Roger adjustment because other methods have coverage <85%.

In summary, low coverage is caused by bias and variability in the SE estimate. REML can be used to reduce bias and the Satterthwaite approximation can be used to account for uncertainty in the SE. These methods improve coverage when complex error models are used. However, such models are still often not desirable because they come at a cost of reduced statistical power.

## 7 DISCUSSION

In our simulation, study we explored the performance of the most commonly used methods for handling autocorrelation in terms of bias, MSE, and confidence interval coverage. Consistent with theoretical results, we found that all methods are unbiased, and at large sample sizes (*n* > 100), there was also little to distinguish the methods—all methods were associated with similar MSE and coverage close to the nominal value. Differences were more apparent at small sample sizes. Here Prais‐Winsten and ARMA were the most efficient methods, particularly at high levels of autocorrelation, and Prais‐Winsten generally had coverage closest to the nominal level, though all methods, including Prais‐Winsten, were associated with some under coverage.

Our findings on efficiency (MSE) are in keeping with asymptotic results and previous simulation studies. It is well known that feasible GLS estimates, which include Prais‐Winsten and maximum likelihood estimates, are asymptotically efficient provided that the autocorrelation structure is correctly specified.^13^ Furthermore, asymptotic efficiency is maintained even when the model is unknown if model selection is done via the AIC.^37^ Surprisingly theoretical results also show that OLS — which does not account for autocorrelation in the estimation of regression parameters — is also efficient asymptotically.^38^ In finite samples, a number of simulation studies have shown that accounting for autocorrelation can improve efficiency,^39^, ^40^ though OLS is probably more efficient at low levels of autocorrelation.^40^

Our simulations suggest that coverage will often be of greater concern than efficiency. An important determinant of coverage is the number of parameters included in the error model. The Prais‐Winsten method is able to achieve coverage close to the nominal value because it is based on an AR(1) error model, that is, there is a single autocorrelation parameter. In ARMA modeling good coverage is facilitated by keeping the number of parameters to a minimum. To a certain degree, this is achieved by using a model selection criterion such as AIC. However, these criteria are designed to minimize out‐of‐sample prediction error not to ensure correct coverage. Furthermore, model selection is data‐dependent and this too can negatively impact on coverage.^41^ Although ARMA modeling offers greater flexibility, our simulation study suggests that there is little advantage in terms of MSE and coverage over assuming an AR(1) model. A further advantage of assuming an AR(1) model is that it produces a produces a single measure of autocorrelation that can be used to compare between studies and guide sample size calculations for future studies.^42^

Although our simulation results suggest that the Prais‐Winsten method achieves reasonable levels of coverage when analyzing time series with as few as *n* = 20 observations, alternative methods may be necessary when analyzing shorter time series. Several simulation studies have shown that estimation via REML rather than maximum likelihood can help to maintain good coverage in short time series, particularly when confidence intervals are based on a t‐distribution with degrees of freedom estimated using the Satterthwaite method.^7^, ^10^, ^27^ These methods can be implemented using software to fit mixed models—for example, the mixed command in Stata. Parametric bootstrapping offers an alternative approach.^8^ Our exploration of the Kenward Roger method, which is similar to REML in conjunction with Satterthwaite degrees of freedom, suggests that this approach can help to improve coverage but that it is important to keep the number of parameters to a minimum (eg, by fitting an AR(1) model) to avoid low power. Unfortunately, REML + Satterthwaite does not maintain good coverage when time series are very short (*n* < 12) and REML often fails to coverage. In these circumstances, the best strategy appears to be to avoid any adjustment for autocorrelation and use OLS.^7^, ^9^

In conclusion, we recommend the Prais‐Winsten method over ARMA modeling and the Newey‐West method for analyzing ITS. For short time series, (*n* < 20) analysts should consider using REML combined with a Satterthwaite degrees of freedom correction or OLS without any adjustment for autocorrelation.

## Supporting information


**Supplementary Figure 1:** Bias as a function of ITS length (n) in 20 autocorrelation scenarios. The scenarios range from lowest correlation in the top left (lag‐1, lag‐2 and lag‐3 correlations of 0.06, 0.02 and 0.01 respectively) to highest correlation in bottom right (lag‐1, lag‐2 and lag‐3 correlations of 0.74, 0.48 and 0.23 respectively)
Supplementary Figure 2: Relationship between coverage and the number of parameters included in the error model. Coverage deteriorates as the number of parameters increases, particularly when *n* ≤ 50, but it can be brought close to the nominal value by implementing the Kenward Roger (K‐R) method (only shown for *n* ≤ 50 because the method is computationally intensive at larger sample sizes)
Supplementary Figure 3: Bias in MA(3) error model parameter estimates obtained via ML and REML (*n* = 20). The cross and horizontal bar denote, respectively, the true value and the median of the estimates
Supplementary Figure 4: Statistical power of methods with >85% coverage. MA(3) with Kenward Roger adjustment consistently has lower power than other methods. Note that at higher levels of autocorrelation only the Prais‐Winsten is included in the comparison because other methods have coverage <85%

## Data Availability

The data and code for these analyses are available at https://github.com/christian‐bottomley/ITS_Autocorrelation.
