# Supplementary material for: In praise of Prais‐Winsten: An evaluation of methods used to account for autocorrelation in interrupted time series
Source: Stat Med. 2023 Feb 1;42(8):1277–88. doi: 10.1002/sim.9669 (PMC10946734; doi:10.1002/sim.9669)

Supplementary Figures for

In praise of Prais-Winsten: an evaluation of methods used to account for autocorrelation in interrupted time series.

Authors

Bottomley C, Ooko M, Gasparrini A, Keogh RH.

Supplementary Figure 1: Bias as a function of ITS length (n) in 20 autocorrelation scenarios. The scenarios range from lowest correlation in the top left (lag-1, lag-2 and lag-3 correlations of 0.06, 0.02 and 0.01 respectively) to highest correlation in bottom right (lag-1, lag-2 and lag-3 correlations of 0.74, 0.48 and 0.23 respectively).

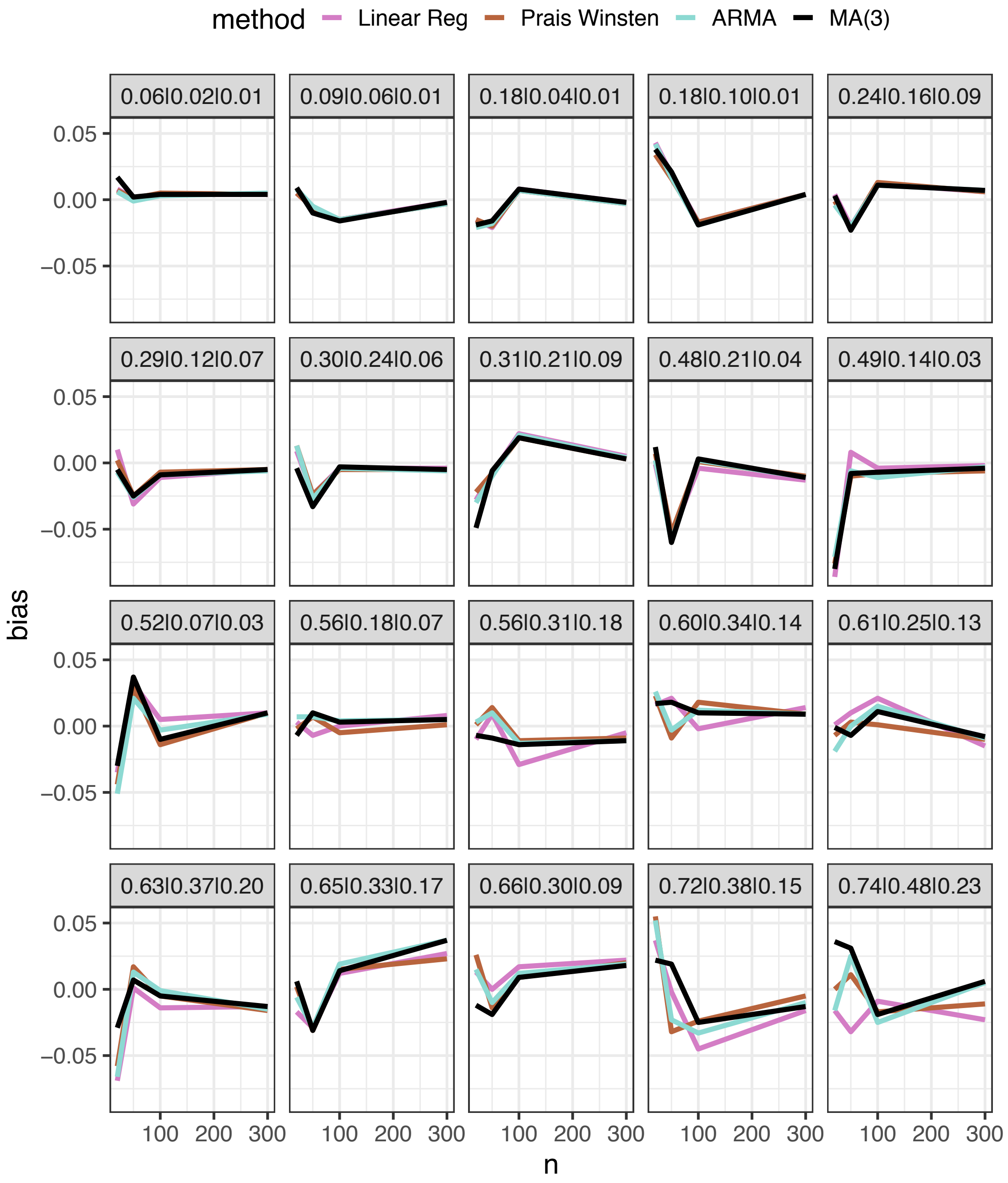

Supplementary Figure 2: Relationship between coverage and the number of parameters included in the error model. Coverage deteriorates as the number of parameters increases, particularly when $n \leq 50$, but it can be brought close to the nominal value by implementing the Kenward Roger (K-R) method (only shown for $n \leq 50$ because the method is computationally intensive at larger sample sizes).

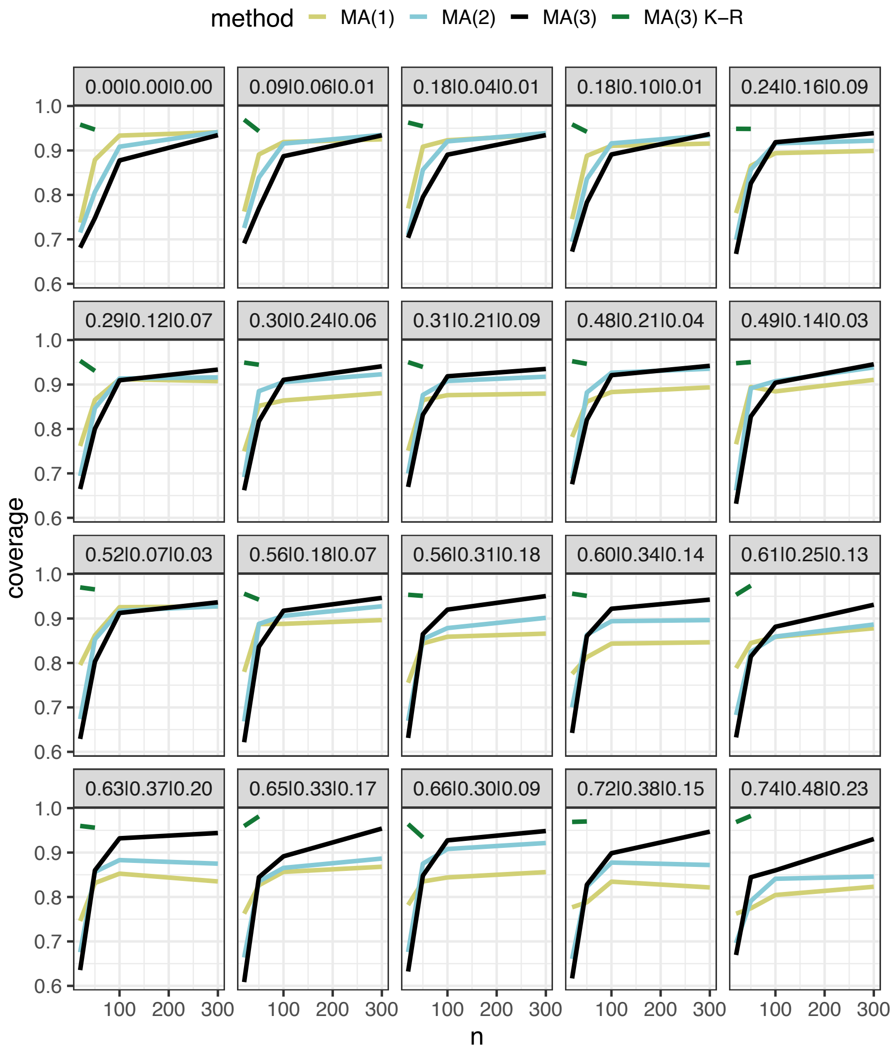

Supplementary Figure 3: Bias in MA(3) error model parameter estimates obtained via ML and REML (n=20). The cross and horizontal bar denote, respectively, the true value and the median of the estimates.

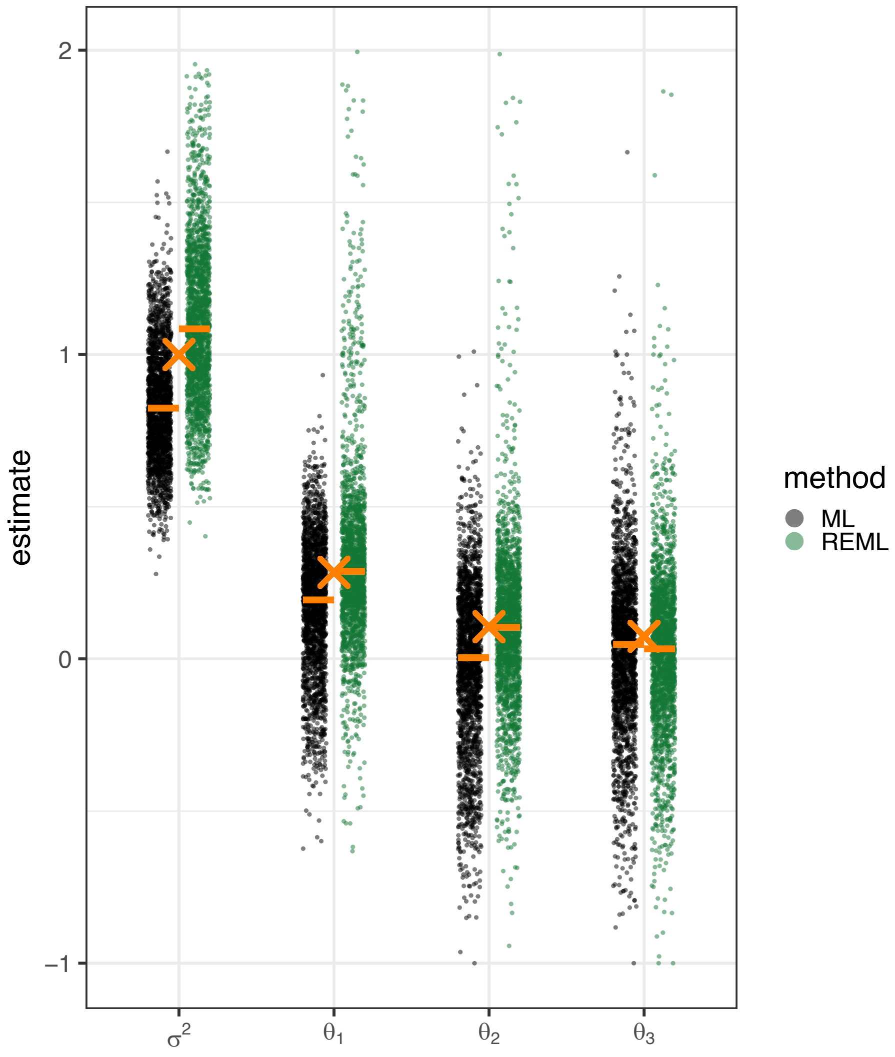

Supplementary Figure 4: Statistical power of methods with >85% coverage. MA(3) with Kenward Roger adjustment consistently has lower power than other methods. Note that at higher levels of autocorrelation only the Prais-Winsten is included in the comparison because other methods have coverage <85%.

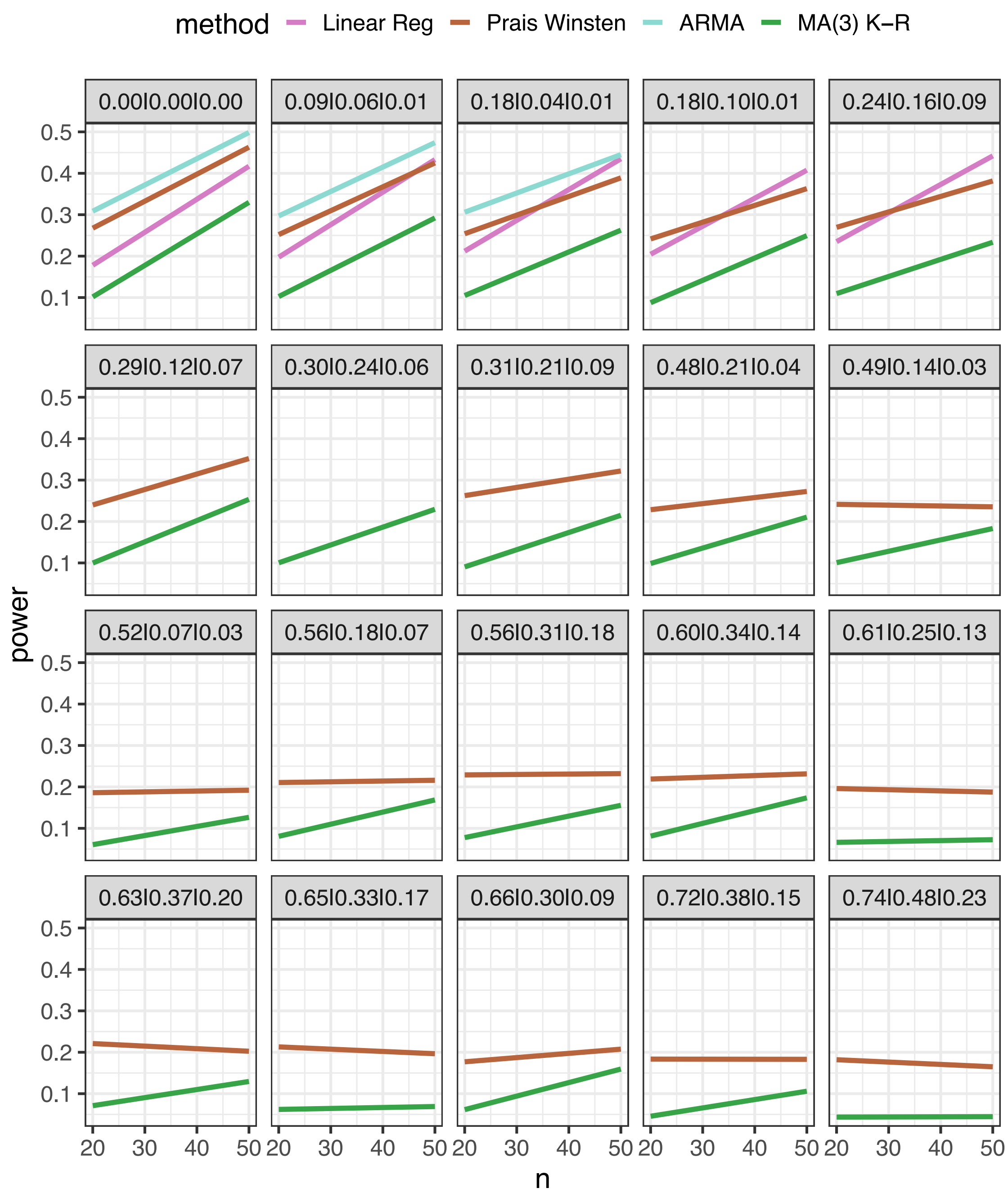

Supplement: Supplementary file 1 — Supplementary Figure 1: Bias as a function of ITS length (n) in 20 autocorrelation scenarios. The scenarios range from lowest correlation in the top left (lag‐1, lag‐2 and lag‐3 correlations of 0.06, 0.02 and 0.01 respectively) to highest correlation in bottom right (lag‐1, lag‐2 and lag‐3 correlations of 0.74, 0.48 and 0.23 respectively) Supplementary Figure 2: Relationship between coverage and the number of parameters included in the error model. Coverage deteriorates as the number of parameters increases, particularly when n ≤ 50, but it can be brought close to the nominal value by implementing the Kenward Roger (K‐R) method (only shown for n ≤ 50 because the method is computationally intensive at larger sample sizes) Supplementary Figure 3: Bias in MA(3) error model parameter estimates obtained via ML and REML (n = 20). The cross and horizontal bar denote, respectively, the true value and the median of the estimates Supplementary Figure 4: Statistical power of methods with >85% coverage. MA(3) with Kenward Roger adjustment consistently has lower power than other methods. Note that at higher levels of autocorrelation only the Prais‐Winsten is included in the comparison because other methods have coverage <85% [file SIM-42-1277-s001.pdf]
